# A high normal TSH level is associated with an atherogenic lipid profile in euthyroid non-smokers with newly diagnosed asymptomatic coronary heart disease

**DOI:** 10.1186/1476-511X-11-44

**Published:** 2012-03-27

**Authors:** Xing Wanjia, Wang Chenggang, Wang Aihong, Yang Xiaomei, Zhao Jiajun, Yu Chunxiao, Xu Jin, Hou Yinglong, Gao Ling

**Affiliations:** 1 Department of Endocrinology, Provincial Hospital affiliated to Shandong University, Jinan, People's Republic of China; 2 School of Public Health, Shandong University, Jinan, People's Republic of China; 3 Department of Preventive Medicine, Shandong University of Traditional Chinese Medicine, Jinan, People's Republic of China; 4 Department of Cardiology, Provincial Hospital affiliated to Shandong University, Jinan, People's Republic of China; 5 Department of Cardiology, Shandong Provincial Qianfoshan Hospital, Shandong University, Jinan, People's Republic of China; 6 Department of Central Laboratory, Provincial Hospital affiliated to Shandong University, Jinan, People's Republic of China

**Keywords:** TSH, Cholesterol, Triglyceride, Coronary heart disease

## Abstract

**Background:**

Serum lipid profiles may be influenced by thyroid function, but the detailed mechanism remains unclear. Increasing evidence suggests that thyrotropin (TSH) may exert extra-thyroidal effects. The goal of this study was to evaluate the relationship between serum TSH levels and the lipid profiles in euthyroid non-smokers with newly diagnosed asymptomatic coronary heart disease (CHD).

**Methods:**

This was a retrospective study of 406 euthyroid non-smokers (187 males and 219 females) with newly diagnosed asymptomatic CHD from 2004 to 2010 in Jinan, China. Lipid parameters and the levels of TSH, FT3, and FT4 were determined. Multiple linear regression analysis and Logistic regression analysis were used to assess the influence of TSH on the lipid profiles and the risks of dyslipidemia.

**Results:**

The TSH level, even within the normal range, was positively and linearly correlated with total cholesterol (TC), non-high density lipoprotein cholesterol (non-HDL-C) and triglycerides (TG) (Beta = 0.173, 0.181 and 0.103, respectively, *P *< 0.01 in all). With 1 mIU/L rise of TSH, the levels of TC, TG and non-HDL-C will increase by 1.010, 1.064, and 1.062 mmol/L, respectively. The odds ratio of hypercholesterolemia and hypertriglyceridemia with respect to the serum TSH level was 1.640 (95% CI 1.199-2.243, *P *= 0.002) and 1.349 (95% CI 1.054-1.726, *P *= 0.017), respectively.

**Conclusions:**

TSH levels were correlated in a positive linear manner with the TC, non-HDL-C and TG levels in euthyroid non-smokers with newly diagnosed asymptomatic CHD. TSH in the upper limits of the reference range might exert adverse effects on lipid profiles and thus representing as a risk factor for hypercholesterolemia and hypertriglyceridemia in the context of CHD.

## Background

In recent years, more and more evidence has demonstrated that hypothyroidism is associated with the increased prevalence of CHD [1,2]. This association is partly due to decreased levels of thyroid hormones, which lead to an atherogenic lipid profile characterized by increased levels of total cholesterol (TC) and low-density lipoprotein cholesterol (LDL-C) [3]. In the past decades, some studies indicate that subclinical hypothyroidism (SCH), which is defined as normal levels of serum free triiodothyronine (FT3) and free thyroxine (FT4) as well as elevated levels of thyrotropin (TSH), is also associated with a moderate increase in the risk of CHD [4,5]. Moreover, some researchers indicate that variations of serum TSH levels within the normal reference range are related to the TC, TG, and LDL-C [6,7], although not all investigations confirm these associations [8,9]. These findings imply that the association between lipid levels and thyroid function cannot be fully explained by the effects of FT3 and FT4 alone. We assumed that TSH, the most sensitive parameter reflecting the abnormal thyroid function in the early stage, might participate in the regulation of lipid profile to some extent.

TSH responds significantly and precisely to minor changes in the concentrations of circulating thyroid hormones. A considerable amount of researches on the possible extrathyroidal effects of TSH has been published. For example, positive associations between the TSH level and waist circumference, body mass index and blood pressure have been described [10-12]. Regarding lipid metabolism, our previous study [13] indicated that TSH might up-regulate hepatic 3-hydroxy-3-methyl-glutaryl coenzyme A reductase (HMGCR) expression, which suggested a potential direct role of TSH in the cholesterol biosynthesis in the liver.

In addition, most studies concerning the relationship between thyroid function and either CHD or the lipid profile had included both smokers and non-smokers. However, it is well known that smoking is also a risk factor for CHD [14]. Furthermore, some population-based study has indicated that smokers have lower levels of TSH [15]. Thus, in this study, we mainly focused on the possible association between TSH and the lipid profiles in euthyroid non-smokers with newly diagnosed asymptomatic CHD.

## Results

### The relationship of TSH and lipid profiles in the study population

Data concerning basic clinical characteristics, including history of diabetes mellitus, history of hypertension, drinking history, TSH, lipid profiles, FBG, UA, positivity of antithyroid antibody, SBP and DBP in the study population were shown in Table 1.

**Table 1:** Clinical characteristics of the study population

characteristic	N = 406
**Male**	187(46.10)
**Age (yr)**	64.38 ± 10.69
**History of hypertension**	290(71.43)
**History of DM**	62(15.27)
**Drinking history**	51(12.56)
**TSH (mIU/L)**	1.81(1.46)
**TC (mmol/L)**	5.05(1.37)
**Non-HDL-C (mmol/L)**	3.73(1.38)
**TG (mmol/L)**	1.40(0.96)
**LDL-C (mmol/L)**	3.00(1.04)
**HDL-C (mmol/L)**	1.26(0.49)
**FBG (mmol/L)**	5.82 ± 1.74
**UA (mmol/L)**	322.46 ± 109.11
**SBP (mmHg)**	135(30)
**DBP (mmHg)**	80(17)
**Antithyroid antibody Positivity**	93(22.91)

Data are presented as means ± standard deviations, medians (interquartile ranges) or number (percentage).

DM, diabetes mellitus; TSH, thyrotropin; TC, total cholesterol; non-HDL-C, non-high-density lipoprotein cholesterol; TG, triglyceride; LDL-C, low-density lipoprotein cholesterol; FBG, fasting blood glucose; UA, uric acid; DBP, diastolic blood pressure; SBP, systolic blood pressure

**Figure 1:**
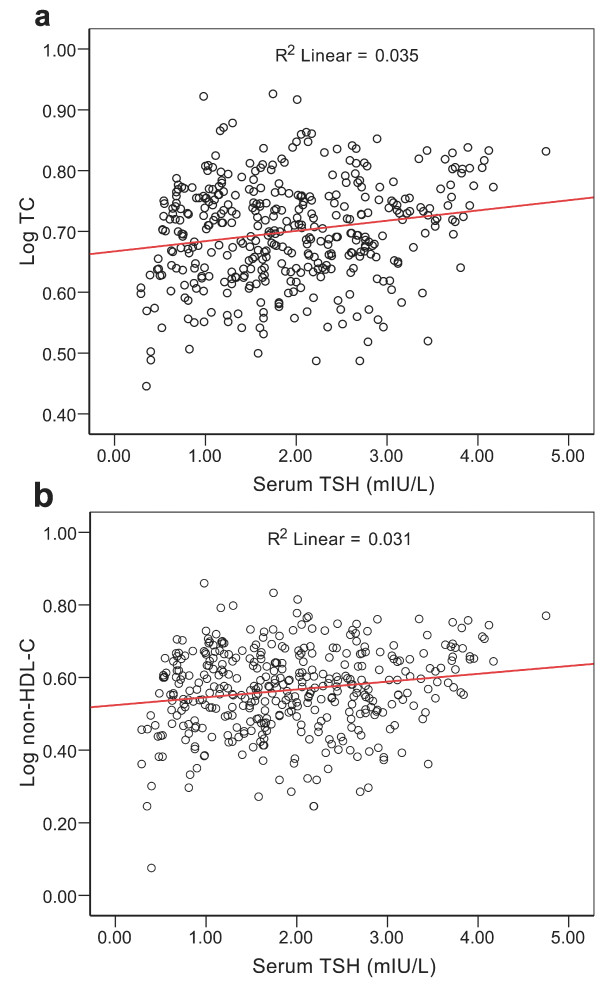
**Positive and linear association between TSH level and log-transformed value of TC (a) or log-transformed value of non-HDL-C (b) in euthyroid non-smokers with newly diagnosed asymptomatic CHD (*P *< 0.01)**.

According to the results of a simple correlation, FT3 and FT4 were not correlated with the log-transformed value of lipid profiles in these subjects. As expected, FT4 was closely and negatively correlated with TSH(r = -0.204, *P *< 0.01) and FT3 was positively correlated with FT4(r = 0.204, *P *< 0.01). Moreover, there were positive linear associations between the serum TSH levels and the log-transformed values of both TC (Figure 1-a) and non-HDL-C levels (Figure 1-b) (*P *< 0.01). TSH level was also positively and linearly associated with log-transformed TG, albeit weak but significant (R^2 ^= 0.011, *P *< 0.05, data not shown). Because FT4 and FT3 are negatively correlated with the TSH level, we did not enter them into the same model in multiple linear regression analysis. Within the normal range, TSH was significantly associated with the log-transformed TC levels (Beta = 0.173, *P *= 0.001), TG levels (Beta = 0.103, *P *= 0.049) and non-HDL-C levels (Beta = 0.181, *P *< 0.001) (Table 2). No evidence was found for an association between the TSH levels and the log-transformed values of HDL-C and LDL-C. In model 2 including FT3 and FT4 in the regression analysis, only FT3 was positively correlated with the log-transformed values of HDL-C (Beta = 0.101, *P *= 0.046). After an anti-log transformation, the results showed that for every 1 mIU/L increase in the TSH levels across the entire reference range, the levels of TC, TG and non-HDL-C will increase by 1.010, 1.064, and 1.062 mmol/L, respectively.

**Table 2:** Multiple linear regression of TSH with lipid profiles in euthyroid non-smokers with newly diagnosed asymptomatic CHD

	Log TC		Log TG		Log non-HDL-C		Log LDL-C		Log HDL-C	
TSH*	**B (95%CI)**	**Beta (P)**	**B (95%CI)**	**Beta (P)**	**B (95%CI)**	**Beta (P)**	**Beta**	**P**	**Beta**	**P**
	0.017(0.007-0.207)	0.173(0.001)	0.027(0.001-0.053)	0.103(0.049)	0.026(0.012-0.041)	0.181(< 0.001)	0.056	0.282	0.018	0.722

Data are B (unstandardized coefficients), 95% confidence interval (CI) of B, Beta (standardized coefficients) and P value for linear regression analysis of TSH, on Log transform of total cholesterol (TC), triglyceride (TG), non-high-density lipoprotein cholesterol (non-HDL-C), low-density lipoprotein cholesterol (LDL-C) and high-density lipoprotein cholesterol (HDL-C).

* adjusted for sex, age, history of diabetes mellitus, history of hypertension, drinking history, fasting blood glucose and uric acid.

### Comparison of lipid profiles among different TSH categories within the normal range

Clinical characteristics of the subjects in different TSH categories were shown in Table 3. Only FBG in G3 and G4 were significantly lower than that in G1 (*P *< 0.05 and *P *< 0.01, respectively). No significant differences existed concerning the levels of FT3, FT4, UA, SBP, DBP and antibody positivity among the four TSH categories.

**Table 3:** Clinical characteristics of different TSH categories in 406 euthyroid non-smokers with newly diagnosed asymptomatic CHD

	G1 TSH 0.3-0.99 mIU/L	G2 TSH 1.0-1.89 mIU/L	G3 TSH 1.9-2.49 mIU/L	G4 TSH 2.5-4.8 mIU/L
**N = 406**	79(19.46%)	135(33.25%)	78(19.21%)	114(28.08)
**Age (yr)**	67.04 ± 10.63	64.59 ± 10.15	63.56 ± 11.32	63.20 ± 10.72
**Male**	40(50.6%)	69(51.1%)	41(52.6%)	37(32.5%)
**DM history**	15(19.0%)	24(17.8%)	12(15.4%)	12(12.3%)
**Drinking history**	4(5.1%)	21(15.6%)	12(15.4%)	14(12.3%)
**Hypertension History**	55(69.6%)	90(66.7%)	60(76.9%)	85(74.6%)
**FT3 (pmol/L)**	4.34 ± 0.75	4.44 ± 0.74	4.26 ± 0.64	4.26 ± 0.69
**FT4 (pmol/L)**	17.73 ± 2.56	17.26 ± 2.25	17.05 ± 2.44	16.38 ± 2.21
**FBG (mmol/L)**	6.26 ± 2.32	6.00 ± 1.82	5.53 ± 1.38*	5.49 ± 1.22**
**UA (mmol/L)**	330.72 ± 112.40	327.80 ± 100.85	314.83 ± 111.46	315.48 ± 115.29
**DBP (mmHg)**	80.00(24)	79.00(17)	80.00(12)	80(16)
**SBP (mmHg)**	135.00(25.00)	132.00(20.00)	132.00(27.75)	135.00(25)
**Antithyroid antibody** **positivity**	16(25.8%)	33(30.6%)	13(19.7%)	31(31.6%)

Data are presented as means ± standard deviations, medians (interquartile ranges) or number (percentage).

DM, diabetes mellitus; FT3, free-triiodothyronine; FT4, free-thyroxine; FBG, fasting blood glucose; UA, uric acid; DBP, diastolic blood pressure; SBP, systolic blood pressure

*P < 0.05 vs.G1; **P < 0.01 vs.G1

Table 4 showed the association between TSH within the normal range and concentrations of serum lipids. The lipid concentrations were log-transformed due to non-normal distribution. Analysis of variance (ANOVA) was performed by LSD (least significant difference) in comparison of lipids in G2, G3 and G4 with that in G1 as the control. There was a consistent and significant increase in concentrations of TC, TG and non-HDL-C, with increasing TSH within the normal range. And more importantly, among the three catrgories (G2, G3 and G4), the log-transformed values of the above lipid parameters increased the most significantly in G4 (TSH 2.5-4.8 mIU/L) than the other two categories when compared with that in G1(*P *< 0.01 in all). While, the log-transformed LDL-C level was only significantly higher in G2 than that in G1 (*P *< 0.05). This trend maintained substantially when G3 and G4 were compared with G1, although not reaching statistical significance (*P *= 0.40 and 0.09, respectively).

**Table 4:** Comparison of log-transformed lipid concentrations between different TSH categories in 406 euthyroid non-smokers with newly diagnosed asymptomatic CHD

	G1 TSH 0.3-0.99 mIU/L	G2 TSH 1.0-1.89 mIU/L	G3 TSH 1.9-2.49 mIU/L	G4 TSH 2.5-4.8 mIU/L
**LogTC (mmol/L)**	0.66 ± 0.10	0.70 ± 0.09**	0.70 ± 0.08*	0.71 ± 0.09**
**LogTG (mmol/L)**	0.09 ± 0.26	0.17 ± 0.22*	0.18 ± 0.24*	0.19 ± 0.24**
**LogHDL-C(mmol/L)**	0.10 ± 0.14	0.09 ± 0.12	0.10 ± 0.13	0.11 ± 0.11
**LogLDL-C (mmol/L)**	0.44 ± 0.11	0.48 ± 0.11*	0.46 ± 0.14	0.47 ± 0.14
**LogNon-HDL-C (mmol/L)**	0.51 ± 0.17	0.57 ± 0.10**	0.56 ± 0.13*	0.58 ± 0.13**

Data are presented as means ± standard deviations.

TC, total cholesterol; TG, triglyceride; HDL-C, high-density lipoprotein cholesterol; LDL-C, low-density lipoprotein cholesterol; Non-HDL-C, non-high-density lipoprotein cholesterol

* *P < 0.0 5 *and * *P < 0.01 vs. G1, respectively

### The relationship of TSH and the prevalence of hypercholesterolemia and hypertriglyceridemia

**Figure 2:**
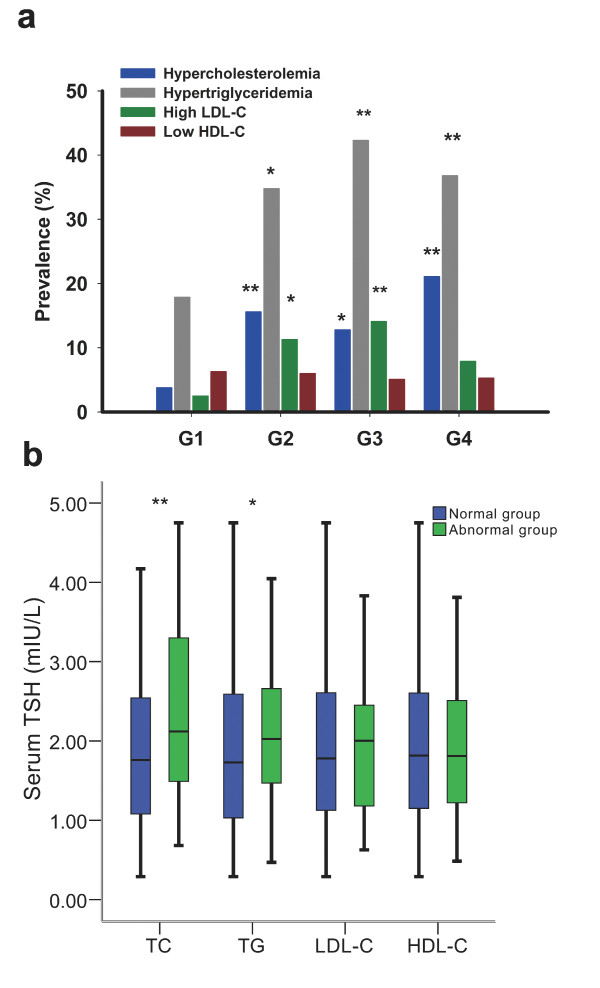
**Association between serum TSH levels and lipid profiles in euthyroid non-smokers with newly diagnosed asymptomatic CHD**. (a) Prevalence of abnormal lipid profiles by categories of different TSH levels within the normal range. *P < 0.05 vs. G1; **P < 0.01 vs. G1 (b) Comparison of serum TSH levels between normal and abnormal groups according to lipid profiles. *P < 0.05 vs. normal group and **P < 0.01 vs. normal group.

The prevalences of hypercholesterolemia, hypertriglyceridemia, high LDL-C and low HDL-C were 14.3%, 33.5%, 9.1%, and 5.7% respectively among the study population. As shown in Figure 2-a, the prevalence of hypercholesterolemia in G2 and G3 was four times higher than that in G1 (*P *< 0.01 and *P *< 0.05, respectively). For hypertriglyceridemia, the prevalence was approximately 2-folds greater in G2, G3 and G4 than that in G1 (*P *< 0.05, *P *< 0.01 and *P *< 0.01, respectively). In G4, the prevalence of hypercholesterolemia was five times higher than that in G1(0.3-0.99 mIU/L). The prevalence of abnormal LDL levels was also higher in G2 and G3 than that in G1 (*P *< 0.05 and *P *< 0.01, respectively). There were no differences in the prevalence of low HDL-C among the four TSH categories.

After adjusting for the confounding factors, logistic regression analysis showed that the TSH level was a significant independent factors for predicting the risk of both hypercholesterolemia and hypertriglyceridemia in the study population. The serum TSH level was not associated with the risk of either high LDL-C or low HDL-C levels in these patients (Table 5).

**Table 5:** Logistic regression of serum TSH level and risk of dyslipidemia in 406 euthyroid non-smokers with newly diagnosed asymptomatic CHD

	B	SE	OR (95%CI of OR)	P
**hypercholesterolemia**	0.494	0.160	1.640(1.199-2.243)	0.002
**hypertriglyceridemia**	0.299	0.126	1.349(1.054-1.726)	0.017
**Low HDL-C**	0.121	0.201	1.129(0.671-1.675)	0.547
**High LDL-C**	0.132	0.257	1.141(0.689-1.890)	0.609

Data are coefficient (B), corresponding standard error (SE), odds ratio (OR) and 95% confidence interval (CI).

Adjusted for sex, age, drinking history, history of diabetes, history of hypertension, fasting blood glucose and uric acids.

HDL-C, high-density lipoprotein cholesterol; LDL-C, low-density lipoprotein cholesterol.

### Comparison of TSH level between different lipid groups

We further compared the levels of serum TSH between the abnormal and normal lipid groups by Mann-Whitney test. The median of TSH was significantly higher in hypercholesterolemic subjects than that in those with normal TC levels (2.12 vs. 1.76 mmol/L, *P *= 0.002). And compared with those had normal TG levels, the median of TSH was also significantly higher in hypertriglyceridemic subjects (2.03 vs. 1.73 mmol/L, *P *= 0.014). There were no differences in the median of TSH levels between the abnormal and normal groups based on the HDL-C and LDL-C levels (Figure 2-b).

## Discussion

It is generally accepted that overt hypothyroidism accelerates the progression of CHD by increasing levels of TC and LDL-C. Whereas controversy still exists as to whether there is an association between SCH and an adverse lipid profile. Moreover, the issue of the association between SCH and CHD remains complex [16,17]. Because there is a consensus that most of the CHD patients should take statins, which might change the lipid profiles, we chose to investigate those with newly diagnosed asymptomatic CHD to avoid the confounding effects of statins on lipid levels. Furthermore, to exclude the possible confounding effects of smoking and to better understand the role of TSH in CHD, we mainly focused on euthyroid non-smokers in this study. We found that even within the normal range, TSH was positively and linearly correlated with lipid profiles in non-smokers with newly diagnosed asymptomatic CHD.

Thyroid hormones play essential roles in a variety of metabolic and developmental processes, and abnormal thyroid hormone metabolism is one of the important causes of coronary artery sclerosis [18]. Besides the direct effects of FT3 and FT4, the relationship between serum TSH concentration and metabolic effects has been the focus of recent studies, which have yielded conflicting results. One study in Japan found that compared with the normal control subjects, CHD patients had a relatively high level of TSH [19]. In a prospective population-based study with a mean follow-up of 10.6 years [20], researchers demonstrated that within the normal range, the TSH level was associated with TC, LDL-C, and HDL-C more strongly than FT4 among both men and women. In the present study, we also found a significant positive linear correlation between serum TSH levels within the normal range and the levels of TC and non-HDL-C. Similarly, a recent large cohort study of Indian women with normal thyroid function [21] also suggested that TSH in the upper limits of the reference range (above 2.5 mIU/L) was associated with higher TC.

In addition, our study showed that TSH was positively correlated with TG, which was also observed in healthy men in Spain [22]. Moreover, a previous study [23] revealed that euthyroid subjects with a TSH level in the high-normal range (2.5-4.5 mIU/L) had higher TG levels than those with TSH levels in the low-normal range (0.3-2.5 mIU/L) (1.583 ± 0.082 vs. 1.422 ± 0.024 mmol/L, *P *= 0.023). We speculated that this association could not be explained by the levels of FBG alone, because the FBG in those with high normal TSH were significantly lower than that in those with TSH in the lower limits of the normal range. The positive association between FT3 and log-transformed values of HDL-C in our study was in accordance with the observations of Ness et al. [24], who found that triiodothyronine could raise HDL-C in rat.

Furthermore, we found that the TSH level was significantly higher in the hypercholesterolemic and hypertriglyceridemic subjects than in patients with normal levels of TC and TG. Similar results have been obtained by Lai et al. [25], who demonstrated that the TSH level in the hypertriglyceridemia group was much higher than in the normal control group. In case of lipid profiles, the concentrations of TC, TG and non-HDL-C were significantly higher in patients whose TSH level were in the upper limits than those whose TSH levels were in the lower limits of the normal range. This phenomenon was supported by the HUNT study [7], which suggested that within the clinically normal TSH range, the increasing level of TSH was associated with less favorable lipid concentrations.

Concerning the risk of dyslipidemia, our data showed that the subjects with relatively high TSH levels within the reference range were more likely to have hypercholesterolemia and hypertriglyceridemia, with the ORs of approximately 1.640 and 1.349. Therefore, according to these results, even within the normal range, subjects with relatively high TSH levels might be prone to dyslipidemia. More attention should be paid to lipid profiles of those patients who had relatively higher serum TSH levels, even within the normal range.

The detailed mechanisms responsible for the effects of TSH on the lipid profile remained unclear. Traditionally, the main function of TSH is to stimulate the synthesis and release of thyroid hormones in the thyroid gland via the specific cell membrane receptor-TSHR. It is now recognized that TSHR is expressed widely in a variety of extra-thyroidal organs including kidney, bone marrow and adipose tissue [26], and act as a physiological regulator in the growth and development of adipocytes [27]. More importantly, emerging evidence suggests that TSH not only acts on the thyroid gland, but also targets on several other organs and tissues. The mechanisms for the regulation of cholesterol homeostasis include effects on biosynthesis, uptake, and metabolism; and the liver is vital for both endogenous cholesterol synthesis and elimination [28]. Previously, our laboratory [29] demonstrated that TSHR is functionally present in both human and rat hepatocytes. A late study [13] revealed that TSH promoted the expression of HMGCR, the rate-limiting enzyme in cholesterol synthesis in liver cells. Based on these findings, we assumed that TSH, even within the normal range, might act through TSHR expressed on hepatocytes to up-regulate the expression of HMGCR resulting in increased TC levels in CHD patients. Moreover, we also speculated that there might be other mechanisms involved in the regulation of lipid profiles by TSH.

To date, the pathophysiological role of TSH *per se *on the cardiovascular system remains unclear. The administration of recombinant human TSH has been reported to be associated with the acute impairment of endothelial function in patients without evidence of functional thyroid tissue [30]. Considering the elevated levels of TC, TG and non-HDL-C and the increased prevalence of hypercholesterolemia among patients whose TSH levels were in the upper limits, we hypothesized that high-normal TSH might have harmful effects on cardiovascular health. Meanwhile, we should also note that all current associations between TSH and lipid profiles have been weak to modest, and thus the detailed mechanism and clinical implications about the effects of TSH on the lipid profile and CHD remains to be elucidated.

The main limitation of this study is its small size. Furthermore, the thyroid status was classified in all patients based on one blood test. Thus, some individuals with transient TSH elevations might have been misclassified. Further studies should involve repeated TSH testing. In addition, with a retrospective study that did not include follow-up, we were unable to determine whether differences in TSH represent an actual risk factor for CHD. In the statistical analysis, the relatively simple multivariate regression models we used explained only a limited amount of variations in the serum lipid profiles. In the future, large-scale studies with longer follow-up peroids will be needed to estimate the real impact of this association and its clinical significance.

In summary, we found that the TSH level was positively and linearly correlated with the TC, non-HDL-C and TG levels, and the prevalence of hypercholesterolemia and hypertriglyceridemia in non-smokers in a Chinese population with newly diagnosed asymptomatic CHD. Our study indicated that even within the normal range, TSH in the upper limits might exert adverse effects on the lipid profile and thus might represent a risk factor for hypercholesterolemia and hypertriglyceridemia in these patients. In the treatment of CHD and dyslipidemia, thyroid function (especially the serum TSH level) should be monitored and maintained in the relatively low-normal range. Further large prospective studies are needed to clarify the above relationship and to confirm its clinical implications.

## Methods

### Study design and sampling

We conducted a retrospective study with subjects from the health centre of Qianfoshan Hospital or Shandong Provincial Hospital (Shandong, China) between January 2004 and September 2010. The study was carried out in compliance with the Declaration of Helsinki.

A total of 921 subjects with newly diagnosed asymptomatic CHD, 45-88 years were involved. The exclusion criteria include: (1) patients without information on vital status or with missing data on serum TSH or thyroxine levels(n = 66); (2) patients taking medications that might affect FT4 or TSH levels or lipid profiles (such as thyroid hormones, anti-thyroid drugs, iodine, amiodarone, estrogens, androgens, steroid hormones, statins, and fibrates)(n = 68); (3) patients who had neurologic diseases, hepatic disorders, renal disorders, or euthyroid sick syndrome which is characterized by abnormal low serum FT3 but normal FT4 and TSH (n = 98); (4) smokers, including both present and past tobacco users (n = 168). Among the remaining 521 non-smokers with newly diagnosed asymptomatic CHD, 406 euthyroid subjects (187 males, 219 females) were recruited as the study subjects.

### Data collection

Data were obtained from medical records. All the patients were stable at the moment of blood samples collection, as none of them were receiving heparin, diuretics or suffering from acute illness, which might influence the accuracy of thyroid hormone assay. The levels of FT3, FT4, TSH, anti-thyroglobulin antibody (anti-TG) and anti-thyroid peroxidase antibody (anti-TPO) were measured by using an electrochemiluminescence immunoassay (Elecsys 2010, Roche, Basel, Switzerland) with intra- and inter-assay coefficients of variation of less than 5%. The laboratory reference ranges for TSH, FT4, and FT3 were 0.27-4.2 mIU/L, 12-22 pmol/L, and 3.1-6.8 pmol/L, respectively. The lipid profile, fasting blood glucose (FBG) and uric acid (UA) level were measured using an automatic biochemistry analyser (OLYMPUS AU5400, Olympus, Japan) and commercial kits. All patients were asked to rest at least 30 minutes and then in a sitting position, the blood pressure of their right arm was measured twice with a desk-model sphygmomanometer. There was a 3-minutes interval between the two measurements for each participant, and the mean value of the two measurements was used as systolic bold pressure (SBP) and diastolic blood pressure (DBP). The rest and exercise stress ECG were assessed by two trained cardiologists who were unaware of the clinical presentation of the patients.

### Diagnostic criteria

(1) Thyroid status was diagnosed according to data previously published in Chinese [31]: Euthyroidism is defined as TSH within 0.3-4.8 mIU/L and FT4 and FT3 within the above laboratory reference ranges. (2) Asymptomatic CHD patients were diagnosed according to the Monica criteria [32]: two or more ECG showing specific changes without suggestive symptoms, and confirmed by the presence of at least one lesion with ≥50% luminal stenosis by coronary angiography. Abnormal ECG findings include the ST segment abnormalities, T wave abnormalities, and other abnormalities indicate myocardium ischemia. (3) Dyslipidemia: Fasting TC > 5.18 mmol/L (200 mg/dl, hypercholesterolemia), fasting TG > 2.27 mmol/L (200 mg/dl, hypertriglyceridemia), fasting HDL-C < 1.04 mmo/L (40 mg/dl, low-HDL-C) in male or < 1.29 mmol/L (50 mg/dl, low-HDL-C) in female, fasting LDL-C > 2.59 mmol/L(100 mg/dl, high LDL-C) [33]. (4) Non-smokers were defined as never daily use of tobacco.

### Grouping of the subjects

Because Teng et al. [31] indicated that a baseline TSH level of 1.0-1.9 mIU/L is an optimal interval to ensure a low incidence of clinical thyroid diseases within five years, and lower limits for normal TSH at 2.5 mIU/L have been suggested based on epidemiological studies [34], we divided those euthyroid patients into four categories: G1(lower limits of TSH, 0.3-0.99 mIU/L), G2(TSH 1.0-1.89 mIU/L), G3(TSH 1.9-2.49 mIU/L) and G4 (upper limits of TSH 2.5-4.8 mIU/L). Furthermore, to compare TSH levels among euthyroid subjects with varying lipid profiles, all subjects were classified as abnormal or normal according to the lipid levels.

### Statistical analysis

Before statistical analysis, the normality of the distribution and the homogeneity of the variances were evaluated using Kolmogorov-Smirnov test and Levene's test, respectively. Data are presented as means ± standard deviations (SD) or medians with interquartile ranges in the case of a skewed distribution. Statistical analysis were performed with ANOVA followed by a multiple-comparison test for subgroups by LSD. The difference of TSH within normal range between normal and abnormal lipid groups was tested by Mann-Whitney test. The log transformation of lipid parameters was performed to correct the skewed distribution [35]. The relationships between sex, age, history of DM, history of hypertension, drinking, TSH, FT3, FT4, FBG, UA and the log-transformed lipid profile were assessed by simple correlation analysis. Stepwise multiple linear regression analysis was performed to estimate the influence of the above parameters on the lipid profile. Because FT4 (FT3) and TSH are closely correlated, we did not use FT4 and FT3 in the same model with TSH. Model 1 includes TSH and other parameters except FT3 and FT4, while model 2 includes FT3, FT4 and other parameters except TSH. The results are expressed as beta (b)- coefficients with 95% confidence intervals (CI) denoting the SD change in terms of lipid profile markers per unit increase in the TSH level. Backward stepwise logistic regression was used to evaluate the odds ratio (OR) of dyslipidemia in relation to serum TSH. The level of statistical significance was set at *P *< 0.05, except for the regression analysis, in which *P *< 0.10 was set as the cut-off point. All the above analyses were conducted with the use of SPSS software (SPSS Inc., Chicago, IL, USA), in version 17.0.

## Abbreviations

ANOVA: analysis of variance; CHD: coronary heart disease; DBP: diastolic blood pressure; FBG: fasting blood glucose; FT3: free-triiodothyronine; FT4: free-thyroxine; HDL-C: high-density lipoprotein cholesterol; HMGCR: 3-hydroxy-3-methyl-glutaryl coenzyme A reductase; LDL-C: low-density lipoprotein cholesterol; LSD: least significant difference; non-HDL-C: non-high-density lipoprotein cholesterol; SBP: systolic blood pressure; SCH: subclinical hypothyroidism; TC: total cholesterol; TG: triglyceride; TSH: thyrotropin; TSHR: thyrotropin receptor; UA: uric acid.

## Competing interests

The authors declare that they have no competing interests.

## Authors' contributions

XWJ conducted the research, analyzed the data and drafted the manuscript. WCG helped in stastical analysis and figures preparation. WAH and YXM participated in data collection. YCX and XJ helped in conducting the research. ZJJ, GL and HYL conceived of the study, participated in the coordination and helped to draft the manuscript. All authors read and approved the final manuscript.
